# Functional Adaptation of the Calcaneus in Historical Foot Binding

**DOI:** 10.1002/jbmr.3185

**Published:** 2017-07-06

**Authors:** Natalie Reznikov, Carina Phillips, Martyn Cooke, Amin Garbout, Farah Ahmed, Molly M Stevens

**Affiliations:** ^1^ Department of Materials Department of Bioengineering and Institute for Biomedical Engineering Imperial College London London UK; ^2^ Imaging and Analysis Centre Core Research Laboratories The Natural History Museum London UK; ^3^ The Hunterian Museum The Royal College of Surgeons of England London UK

**Keywords:** FOOT, BONE ADAPTATION, INTER‐TRABECULAR ANGLE, MICROCT, STRUCTURE‐FUNCTION RELATIONSHIP

## Abstract

The normal structure of human feet is optimized for shock dampening during walking and running. Foot binding was a historical practice in China aimed at restricting the growth of female feet for aesthetic reasons. In a bound foot the shock‐dampening function normally facilitated by the foot arches is withdrawn, resulting in the foot functioning as a rigid extension of the lower leg. An interesting question inspiring this study regards the nature of adaptation of the heel bone to this nonphysiological function using the parameters of cancellous bone anisotropy and 3D fabric topology and a novel intertrabecular angle (ITA) analysis. We found that the trabecular microarchitecture of the normal heel bone, but not of the bound foot, adapts to function by increased anisotropy and preferred orientation of trabeculae. The anisotropic texture in the normal heel bone consistently follows the physiological stress trajectories. However, in the bound foot heel bone the characteristic anisotropy pattern fails to develop, reflecting the lack of a normal biomechanical input. Moreover, the basic topological blueprint of cancellous bone investigated by the ITA method is nearly invariant in both normal and bound foot. These findings suggest that the anisotropic cancellous bone texture is an acquired characteristic that reflects recurrent loading conditions; conversely, an inadequate biomechanical input precludes the formation of anisotropic texture. This opens a long‐sought‐after possibility to reconstruct bone function from its form. The conserved topological parameters characterize the generic 3D fabric of cancellous bone, which is to a large extent independent of its adaptation to recurrent loading and perhaps determines the mechanical competence of trabecular bone regardless of its functional adaptation. © 2017 The Authors. *Journal of Bone and Mineral Research* Published by Wiley Periodicals Inc.

## Introduction

Loading is known to augment bone,^1^ whereas disuse leads to bone loss.^2^ At a less dramatic scale, the parallel processes of bone augmentation and loss via remodeling take place throughout life and arguably drive functional adaptation of the skeleton—the process of simultaneous optimization of mechanical performance and reduction of the metabolic cost. A vision that bone form is a response to external stress was postulated more than a century ago.^3^, ^4^ Since then, much effort has been invested into exploring causal relationships between the mechanical input and the morphological output,^5^, ^6^, ^7^, ^8^, ^9^ with most investigators agreeing that such a connection should be interpreted with caution (for an excellent review see Kivell^10^). What is more certain for the skeleton, is that functional adaptation of bone starts from a common, generic blueprint of bone architecture that is morphologically consistent, nonspecialized, and adaptable.^9^ Early in life, an adaptable default structure must already be mechanically competent, to allow subsequent adaptation for survival. Obviously, a big challenge in deciphering an individual adaptation trajectory is to deconvolve the plethora of possible biological and biomechanical variables (eg, biological profile, activity level, and health status) to which specific morphological features of bone can be confidently attributed.

**Figure 1:**
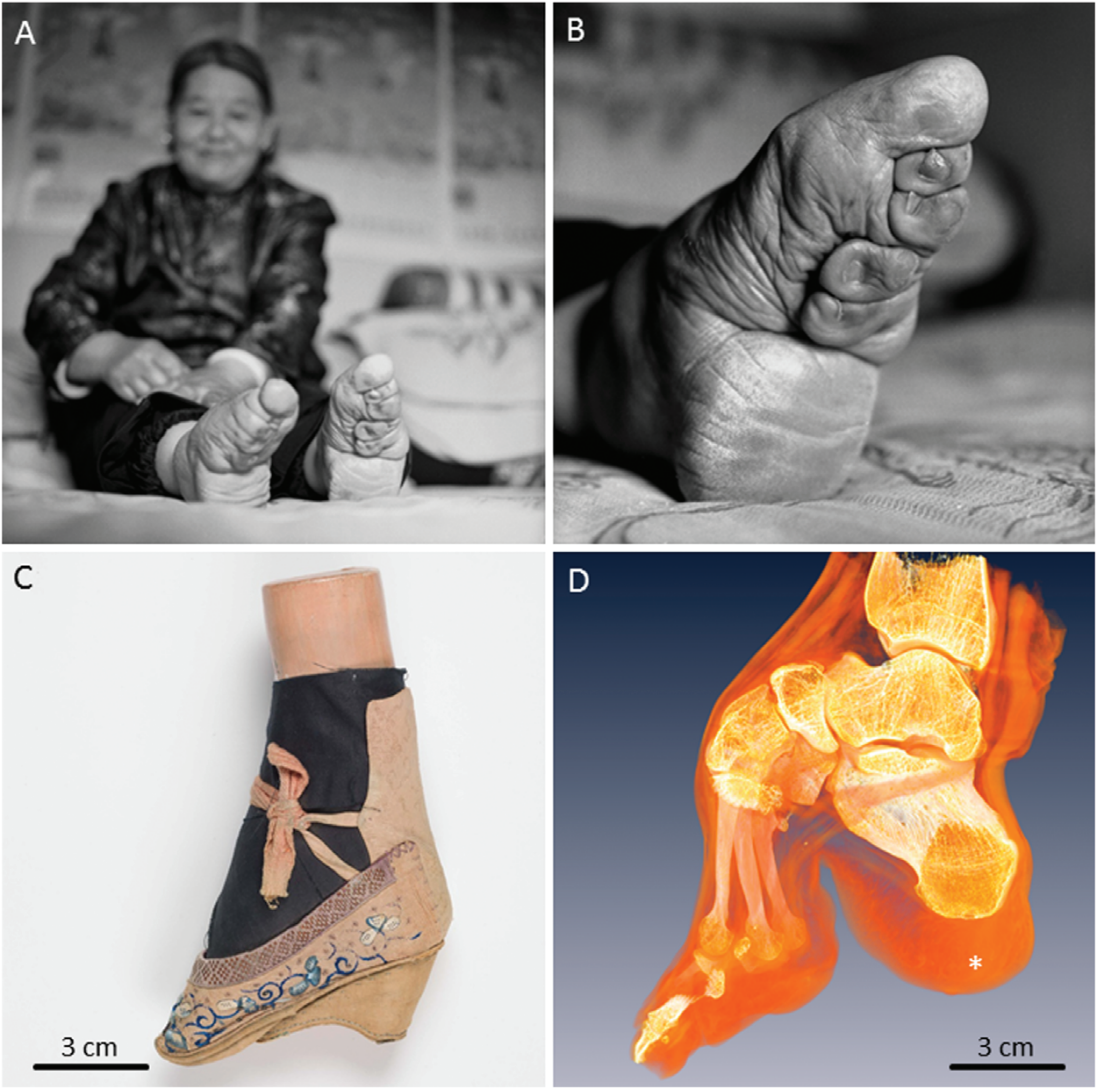
Golden lotus: cultural skeletal deformation of the foot. A woman with bound feet (*A*) and a close‐up view of the underside of her left foot (*B*). Su Xi Rong had her feet bound at the age of 7 years, and she was thought to be the most beautiful woman in the village because of her small and well‐formed feet. Courtesy of Jo Farrell (Jo Farrell Photography, http://www.livinghistory.photography/). (*C*) An embroidered “Golden Lotus” shoe occasionally worn by a woman having bound feet (courtesy of the Museums at the Royal College of Surgeons, London, UK). (*D*) Three‐dimensional reconstruction by μCT of another bound foot showing anatomical structure and relationships for both skeletal elements (yellow) and soft tissues (orange). Specimen RCSPC/02081. Asterisk indicates the increased thickness of the adipose tissue pad directly under the calcaneus (also apparent in *B*).

We hypothesized that if physiological loading of the foot bones indeed gradually shapes their trabecular organization via remodeling, then prolonged abnormal loading of the foot would also change trabecular three‐dimensional architecture. In this context, a comparison was made between the trabecular parameters of the heel bone (calcaneus) in normal human feet and in the extraordinary historical example of foot deformation as performed in China (Fig. 1A, B, C). Whereas in both cases the calcaneus is a functional component of the locomotory system, and its original anatomical form (ie, the default blueprint) is the same, in the case of the bound foot the mechanical input is dramatically altered from childhood, suggesting that the morphological output of adaptation will also diverge from the norm. In particular, we considered how the loss of the shock‐dampening role of the foot arches affects the trabecular architecture.

### Biomechanics of the normal foot: form follows function?

**Figure 2:**
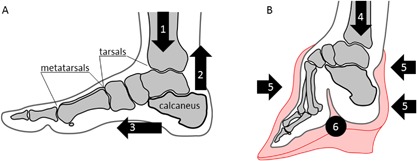
Comparison of the anatomy and the distribution of load in normal (*A*) and bound (*B*) feet. (1) Vertical loading of the normal foot in a broad range; (2) vertical pull of the Achilles tendon; (3) horizontal pull of the plantar fascia; (4) vertical loading of the bound foot in a limited range; (5) compressive force of the bandage; (6) folded, narrowed longitudinal arch of the bound foot.

Human feet are impressively effective structures, allowing upright walking and attenuation of impact during bipedal locomotion. A human‐like longitudinal arch in the foot may have evolved as early as 4 million years ago, following commitment to bipedalism.^11^, ^12^ One quarter of our bones are in the feet; of the 26 bones forming each foot, the seven tarsals (talus, calcaneus, three cuneiforms, cuboid, and navicular bones) and the five metatarsals are arranged into the longitudinal arch, the ends of which are pulled together by the plantar fascia so that the two abutments of the arch are the calcaneus and the distal ends of the metatarsal bones^13^ (Fig. 2A). The talus, forming the summit of the arch, articulates with the lower leg and conveys the load of body weight towards the resilient assembly below. The Achilles tendon is inserted into the calcaneus posteriorly, and is toned by the lower leg muscles. In this anatomical arrangement, the calcaneus articulates proximally with the other tarsals at the fulcrum, and its distal end is balanced between the tendon and the fascia. The calcaneus bears more than 40% of body weight and contributes to the shock‐dampening structure of the foot, where elements in compression (the bones) are counteracted by elements in tension (tendons, fasciae, and ligaments).^13^

### Historical practice of foot binding

Foot binding was a cultural practice that caused skeletal deformation of the feet.^14^, ^15^ This procedure was performed in Han China since the XIth century, and was banned by the Chinese government in 1912. Foot binding was conducted only on females, and its purpose was to restrain growth and reshape the feet in order to meet certain aesthetic criteria—the tiny “golden lotus” foot appearance was considered to be attractive (Fig.1 C). Although started by Chinese nobility, by the XVIIth century the tradition had spread across all social strata for the sake of enhancing matrimonial prospects. As opposed to higher‐status bound‐feet individuals who had continuous assistance in their everyday routines, those in rural areas had little alternative but to adapt to their condition and move around independently. Testimonials from the last survivors of foot binding confirm that they were not only able to walk, but also able to dance, ride a bicycle, and even bowl.^16^ Technically, the procedure of foot binding involved the following: toes II to V (except the big toe) were hyperflexed and curled under the sole, and thus they were excluded from supporting the foot. Then, the metatarsals were rearranged (folded) into a narrowed steep “arch,” and the calcaneus was gradually aligned with the long axis of the lower leg (Fig. 2B) by means of a bandage pressure. The optimal age for foot binding was considered to be between 4 and 7 years old, the age at which the primary centers of ossification are present in the tarsal bones, the secondary centers of ossification appear in the metatarsals, and the foot arch is forming.^17^ A bound‐foot young girl could eventually walk unassisted in a peculiar, characteristic short‐stride manner, a gait which was considered aesthetically gratifying. The foot deformity persisted for a lifetime.

### Biomechanics of the bound foot

The range of motion in the ankle of a bound‐foot female is reduced by at least 60%.^18^ The center of mass is shifted posteriorly from the forefoot to the calcaneus, which is reoriented by about 40 degrees sagittally with respect to the normal anatomical position (Fig. 2). Although the longitudinal arch in a bound foot looks significantly steeper than its normal shape, it is externally compressed by the applied bandage, instead of being a part of the natural tension‐compression assembly. The shock‐dampening function of the foot in this case is essentially eliminated, with the calcaneus acting as a rigid extension of the lower leg and bearing most of the body weight. Despite this profound skeletal deformation, the short bones of the foot remain mechanically competent; among many reported adverse effects of foot binding, such as excruciating pain, circulation impairment, balance problems, infections, and others, fractures of the calcaneus have never been mentioned.^14^, ^15^

### Structure and function of cancellous bone tissue: a shock‐dampening microarchitecture

The role of trabecular tissue is now known to be far more than merely a lightweight filler of bones.^19^ The importance of cancellous bone tissue is highlighted by the many significantly loaded anatomical elements that have only a thin compact shell, such as vertebrae, articulating ends of long bones, and the short bones of the hands and feet. Being situated at the sites experiencing multidirectional loading because of their mobility, cancellous bone provides a shock‐dampening system at the tissue level. This follows from its lower apparent modulus^20^, ^21^ in comparison to that of compact bone. In a previous study of cancellous bone topology (the manner in which individual elements connect to form a continuous three‐dimensional framework) it was suggested by Reznikov and colleagues^22^ that the topological parameters of trabecular (cancellous) bone in the human proximal femur are optimized for collective load bearing and stress redistribution. The findings of that study can be summarized as follows: (1) trabeculae connect at nodes of relatively low valence (ie, the number of struts connected at a node), and (2) the values of the angles between connected trabeculae (the intertrabecular angle [ITA]) center around the angular values characteristic of geometrically regular, symmetrical shapes. This means that in cancellous bone, osseous tissue is sparingly distributed in three‐dimensional space in such a way that the largest volume possible is spanned. Furthermore, the strategy of coping with multidirectional stresses by redistributing them among all structural elements is evident from the organization of cancellous bone at the micrometer scale. At this level, ordered arrays of collagen alternate their orientation either in the direction of their own trabecula or in the direction of one of contiguous trabeculae.^23^ Moreover, the microheterogeneity and inherent prestress of bone material also contribute to shock‐dampening.^24^, ^25^ To infer a general principle from this, the shock‐dampening function is implemented at the anatomical level (eg, foot arches), at the tissue level (trabecular bone topology), and at the material level (co‐alignment of collagen fibrils, and prestress). Hence, to facilitate human locomotion, the shock‐dampening organization is a hierarchical structural phenomenon present across length scales ranging from meters to micrometers. In the present study, we investigate the functional adaptation of cancellous bone in the calcaneus of normal and bound feet by analyzing tissue anisotropy and fabric topology with special attention given to the shock‐dampening function of the foot.

## Materials and Methods

### Foot specimens

**Figure 3:**
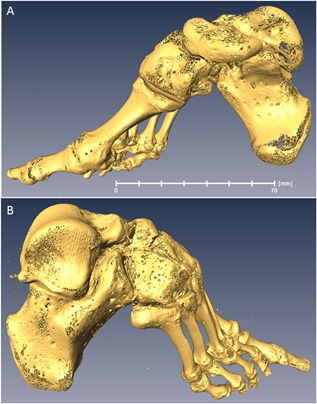
Skeletonized bound foot (BFS110 in the text). Sample RCSPC/02080, Museums at the Royal College of Surgeons, London, UK. Acquired in 1858. Full scan, pixel size 0.072 mm; calcaneus, pixel size 0.037 mm. (*A*) Medial aspect. (*B*) Lateral aspect. Estimated skeletal age ca. 13 years. A three‐dimensional surface scan of this specimen can be viewed here: http://www.digitiseddiseases.org/mrn.php?mrn=R1030.

**Figure 4:**
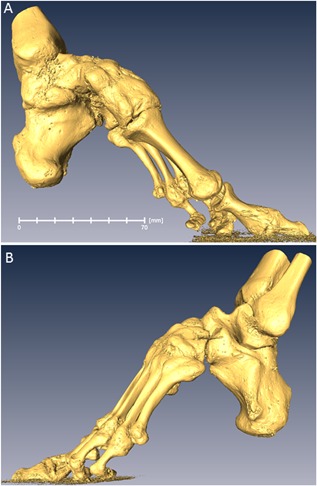
Skeletonized bound foot (BFS140 in the text). Sample BPM_TE229, Bart's Pathology Museum, Queen Mary University of London, London, UK. Acquired in the mid‐1850s. Full scan, pixel size 0.085 mm; calcaneus, pixel size 0.041 mm. (*A*) Medial aspect. (*B*) Lateral aspect. Estimated skeletal age: adult.

**Figure 5:**
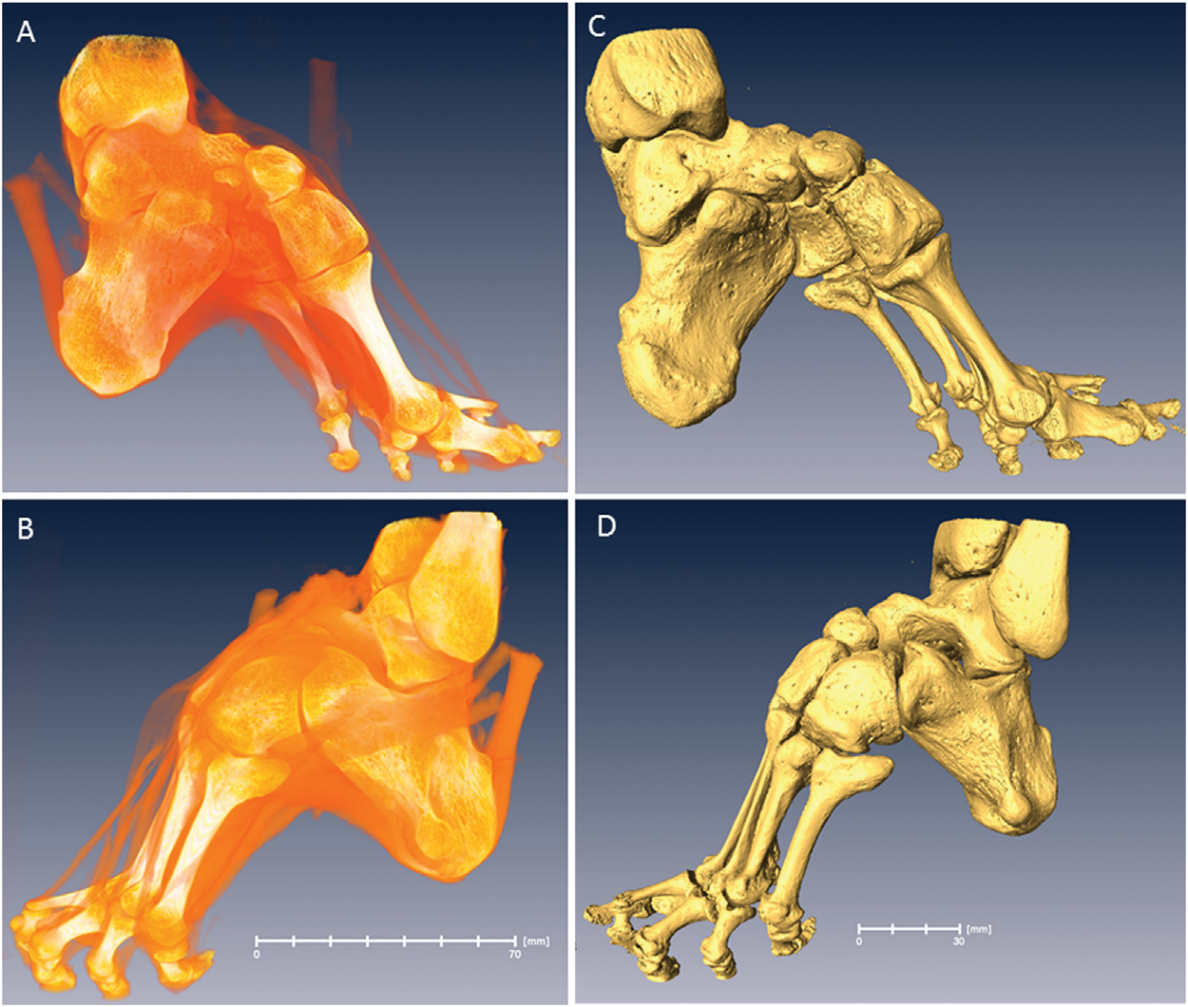
Bound foot with vestiges of tendons and ligaments (BFW132 in the text). Specimen RCSPC/02077, Museums at the Royal College of Surgeons, London, UK. Acquired in 1858. Full scan, pixel size 0.078 mm; calcaneus, pixel size 0.033 mm. (*A*) Medial aspect. (*B*) Lateral aspect. (*C*) Medial aspect, rendering of skeletal structure. (*D*) Lateral aspect, rendering of skeletal structure. Estimated skeletal age: adult.

**Figure 6:**
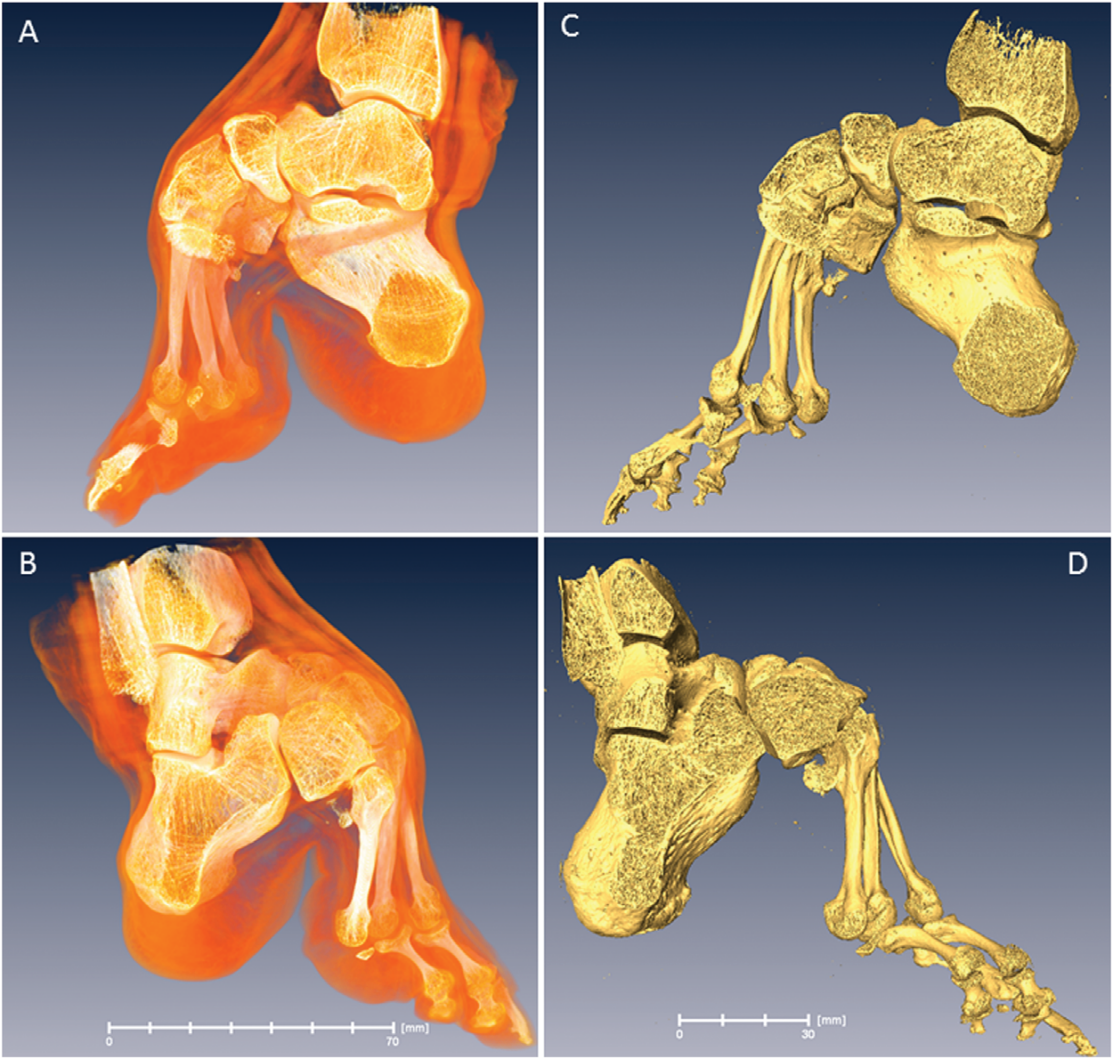
Bound foot with soft tissues (BFW115 in the text). Specimen RCSPC/0_2082, Museums at the Royal College of Surgeons, London, UK. Acquired in 1858. The foot is cut into three pieces in the sagittal direction. All sections are conserved separately and the central one was used in the study. Full scan, pixel size 0.068 mm; calcaneus, pixel size 0.027 mm. (*A*) Medial aspect. (*B*) Lateral aspect. (*C*) Medial aspect, rendering of skeletal structure. (*D*) Lateral aspect, rendering of skeletal structure. Estimated skeletal age: early young adult.

In total, four bound‐foot specimens were selected for this study: two wet, and two skeletonized specimens (Figs. 3(A, B), 4 (A, B), 5 (A–D), 6 (A–D) ). One of the wet specimens had been sagittally cut into three pieces exposing the inner structure of the bones and ligaments; the three pieces of one foot were sealed in individual containers. As per standard current conservation practice, the wet specimens are mounted in acrylic containers and stored in a solution of Kaiserling III or in liquid paraffin. To reduce X‐ray attenuation, and to increase contrast during X‐ray scanning of the feet, it was necessary to decant the preservative solutions from the containers (and then to re‐fill the containers afterward for continued preservation). Three of the bound‐foot specimens came from the museum collections at the Royal College of Surgeons of England (RCS). These preparations were originally acquired by Naval surgeon Stephen Stanley. Stanley served in the Anglo‐Chinese War of 1842, also known as the Opium War, where it is possible that he collected these preparations. The specimens were donated to the RCS by his widow in 1858. The fourth (skeletonized) specimen came from Bart's Pathology Museum, Queen Mary University London, and also dates to the middle of the XIX century. The precise geographical origin of the specimens within China, and the age at which foot binding was conducted in these cases, are unknown. Nevertheless, the reduction in the longitudinal dimension and the alteration of the foot bones’ alignment are remarkable and cannot be mistaken. The bound‐foot specimens all had well‐preserved trabecular architecture despite any inevitable biochemical degradation of the organic constituents. For comparison, six morphologically normal, assembled skeletal foot specimens were also used in the study. The normal samples ranged in length between 197 to 229 mm, suggesting that they were likely female.^26^, ^27^ The normal samples, Supporting Fig.  1, were provided by the Anatomy Department, Faculty of Medicine, Imperial College London (teaching collection at Charing Cross Hospital). All the museum and teaching specimens used in this study were procured with full compliance with the Human Tissue Act 2004 of the United Kingdom, and ethical permissions were obtained (Imperial College Healthcare Tissue Bank approval no. 12/WA/0196).

### Imaging

X‐ray micro–computed tomography (μCT) was conducted at the Imaging and Analysis Centre, Natural History Museum, London, UK, using a Nikon Metrology HMX ST 225 scanner (Nikon, Tring, UK). The images were captured in 3142 projections using a 2000× (2K X) flat panel detector. The scanning parameters and conditions used were the following: tungsten anode, 0.25 to 0.5 mm copper filter, 700 to 1000 ms exposure time, tube peak voltage 180 kV, and current at 170 mA. The pixel size in the calcaneus tomograms varied between 0.027 to 0.033 mm (bound foot group) and 0.033 to 0.042 mm (control group), depending on specimen size (Supporting Fig.  2B). The focused calcaneus scans were used for analysis. In addition, the museum bound‐foot specimens were scanned in full with a pixel size of 0.07 to 0.085 mm (Supporting Fig.  2A). The full‐size scans were used for illustrative purposes. Reconstruction was carried out using a Feldkamp back projection algorithm with software CTPro (Nikon, Tring, UK).

### Analysis using conventional texture parameters and novel topology descriptors

Following reconstruction to form three‐dimensional images, the trabecular interior of each calcaneus was digitally separated from the compact shell (Amira Software, version 5.3.2; FEI, ThermoFisherScientific, Hillsboro, OR, USA; Supporting Fig.  2) by manually applying irregular anatomical contour a few pixels inward from the compact bone tissue. The trabecular material was segmented using a single‐threshold method (Amira), and the image was converted into an 8‐bit binary format (ImageJ/Image/Adjust/Threshold function; ImageJ software, NIH, Bethesda, MD, USA; https://imagej.nih.gov/ij/) so that osseous tissue was white (255 pixel value) and background was black (0 pixel value). The threshold gray value for creating a binary image was adjusted individually for each specimen within the range 90 to 100 in such a way that the trabecular network appeared continuous throughout the volume (above the pixel value of ∼90) and there were no areas of speckled noise in the intertrabecular spaces (below the pixel value of ∼100). We carried out two types of trabecular microarchitecture analysis: conventional texture examination and fabric topology analysis.

#### Conventional analysis

**Figure 7:**
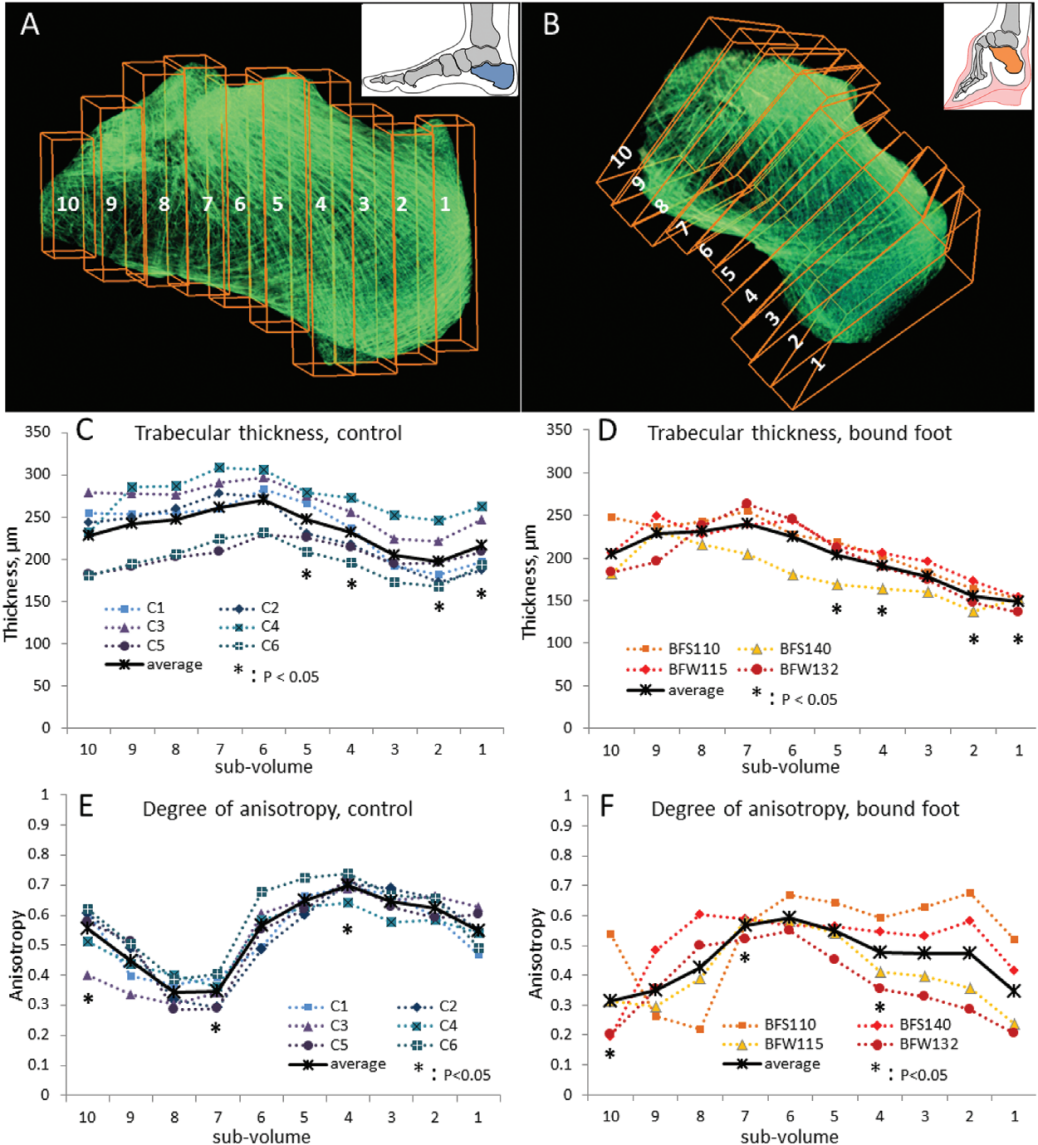
Conventional morphometric analysis of trabecular bone. Ten subvolumes along the longitudinal axis of the normal and bound foot calcanei (*A*, *B*) were used for estimating the general trend of the trabecular thickness (*C*, control calcaneus; *D*, bound‐foot calcaneus) and the anisotropy index (*E*, control calcaneus; *F*, bound‐foot calcaneus). Trabecular thickness and anisotropy were calculated for each subvolume and plotted as individual measurements against the subvolume number. Average values are plotted as a black trend line.

For the conventional analysis, BoneJ/ImageJ (Fiji) data processing software was used.^2^ Each calcaneus was digitally divided into 10 subvolumes along the longitudinal axis using the Image/Stack/Split stack application in ImageJ. The subvolumes were numbered from 10 (at the subtalar joint area) to 1 (the distal‐most part of the calcaneus). The subvolumes within one sample were of identical thickness, around 3 mm thick in the smaller bound feet, and around 4 mm thick in the control feet (Fig. 7 *A*, *B*). For the series of subvolumes we calculated the degree of anisotropy (DA), bone volume fraction (BV/TV), trabecular thickness (Tb.Th), and fractal dimension (FD). Degree of anisotropy is a measure of preferential orientation of substructures in a volume (http://bonej.org/anisotropy). For the bone volume fraction, the calculation was done after lateral cropping of the subvolumes to fit the cropped volume entirely within osseous tissue (Fig. 7 *A*, *B*). No other subvolume normalization was conducted. The values of the parameters Tb.Th, DA, and BV/TV were plotted as individual measurements against the sub‐volume number; the mean values were calculated and plotted as a trend line. Statistical significance of the differences in average values per subvolume was calculated using a *t* test (two‐tailed, unpaired), and statistically significant measurements were indicated with asterisks on the plots.

#### Topological analysis

**Figure 8:**
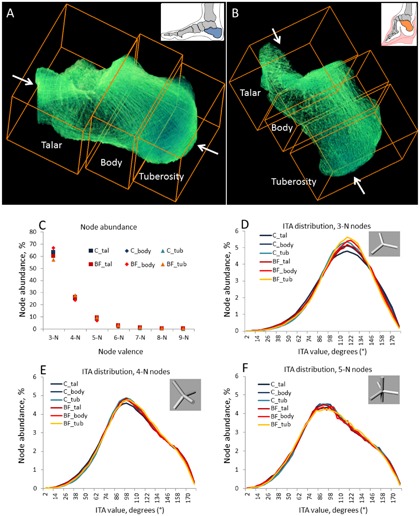
Topological characterization of trabecular bone. ITA analysis of normal (*A*) and bound‐foot (*B*) calcanei shows striking similarity of the node abundances (*C*) and ITA values distributions for each node type (*D*–*F*). Each sample was split in three areas (as shown in *A* and *B*) and then the same areas of different samples were pooled together.

The topological analysis was carried out using a previously published algorithm of statistical evaluation of the nodes’ abundance and the distribution of ITA values.^3^ Briefly, this method evaluates the way in which individual trabeculae of cancellous bone connect with each other throughout an entire anatomical element. Each trabecula in a binary image is digitally replaced by a 1‐pixel‐thick line aligned with the long‐axis of the trabecula (this operation is called digital skeletonization; Supporting Fig.2D, E). These lines, or edges, form a three‐dimensional interconnected fabric with the same orientation and connectivity of the edges as that of the trabeculae of the original bone specimen. The edges are connected at so‐called nodes, and the three‐dimensional angles between the edges are calculated and plotted per each node category of a given valence (3‐N, 4‐N, and 5‐N). The ITA values are plotted as a statistical distribution of tens of thousand and hundreds of thousands of measurements. For the ITA analysis, each calcaneus specimen was digitally divided into three subvolumes, referred to as talar area (proximally from the sustentaculum tali), tuberosity (distally from the bursal projection), and body (in between), Figure 8 A and B. The division into three subvolumes was done perpendicular to the long axis of the calcaneus. Each subvolume generated 200,000 to 300,000 ITA values.

## Results

### Conventional analysis of the trabecular microarchitecture

The internal microarchitecture of the calcaneus is rather heterogeneous, because its different anatomical areas are subjected to different local biomechanical stimuli. Therefore, we present the characteristics of cancellous bone tissue in subvolumes along the longitudinal axis of the calcaneus, starting from the tuberosity (subvolume 1) and ending at the subtalar (talocalcaneal) joint surface (subvolume 10) (Fig. 7A, B). Figure 7 (C‐F) compares the trabecular thickness (panels C and D) and anisotropy (panels E and F) trends in normal and bound‐foot calcanei along their axes, as analyzed using BoneJ.^28^ Whereas in both groups the thickness of trabeculae is generally higher in the proximal area (near the talocalcaneal joint) and slightly decreases distally, we found that trabecular thickness in the bound‐foot group is significantly lower in the distal part of the calcaneus, especially in the most distal segment of the tuberosity area. The next two panels, Fig. 7 *E*, *F*, compare the trend line of the degree of anisotropy along the anatomical axis of the calcaneus. Generally, anisotropy illustrates the extent of preferred orientation of substructures within the entire structure^29^ and ranges between 0 (random, or isotropic, orientation) and 1 (absolutely anisotropic; ie, all structural members exhibit identical orientation). Cancellous bone anisotropy presumably reflects a persistent mode of loading: individual trabeculae with time acquire a certain preferred orientation, which is generally aligned with the stress trajectories as the result of lifelong remodeling. Indeed, even upon visual evaluation, the enhanced preferred orientation of trabeculae in the distal part of the normal calcaneus can be seen as distinctive crosshatching on the tomogram (Fig. 7 *A*) in the directions corresponding to the orientations of the Achilles tendon and the plantar fascia pull (subvolumes 1 to 4). The proximal area of the normal calcaneus, especially corresponding to the subvolumes 7, 8, and 9, is visually more transparent and less organized into stress trajectories. This pattern is strikingly recurring in all the normal calcanei: note the close clustering of the individual measurements in Fig. 7 *E* and the general S‐shape of the anisotropy trend line. In the bound‐foot group, one specimen, BFS110, displays an anisotropy pattern similar to that of the norm, although the other three specimens apparently fail to establish a recognizable anisotropy pattern and rather display an inverse trend (higher anisotropy in the central part of the calcaneus and a drop in anisotropy values toward the anterior articular surface, and even more so toward the tuberosity). Interestingly, these observations of different anisotropy patterns and trabecular thickness are present in the context of nearly identical bone volume fraction values. The bone volume fraction trend lines are plotted against the subvolume number in Supporting Fig.  3. The fractal dimension measured along the calcaneus axis was very similar in both groups with statistically significant but minor decrease in subvolumes 7 and 8 of the control group (Supporting Fig.  4).

### ITA evaluation of the basic topological blueprint of the trabecular fabric

The method of investigation of cancellous bone topology using ITA analysis was developed by Reznikov and colleagues^22^ in 2016. The ITA analysis is blind to the thickness of individual trabeculae and their orientation with respect to the global axes and anatomical landmarks, but rather evaluates the basic topological blueprint of cancellous bone. Each distribution of the ITA values for a given node category is a normal distribution characterized by its mean and width (with the standard deviation being close to the half‐width at half‐maximum [HWHM]), rather than by a single value. Importantly, both the mean and HWHM values of the ITA distribution are also conserved between anatomical sites, implying that even if some nodes deviate from the idealized symmetrical configuration, a certain degree of uniformity is required to render trabecular bone fabric space‐filling. Figure 8 illustrates the ITA parameters for three anatomical areas of the calcaneus. Figure 8 *C* shows the abundance of nodes of different valence that form a continuous three‐dimensional fabric, as a percent of the total number of fabric‐forming nodes. The proportions of the simplest 3‐neighbor (3‐N) and more complex 4‐neighbor (4‐N), 5‐neighbor (5‐N), and other nodes are essentially the same among different anatomical areas of the calcaneus. Moreover, no distinction can be made between the normal and the bound‐foot groups. Figure 8 *D*–*F* show the ITA distributions for each node type (simplified node types are shown in the insets, 3‐N nodes in Fig. 8 D, 4‐N nodes in Fig. 8 E, and 5‐N nodes in Fig. 8 F). From the close overlap of the plotted values it follows that no significant difference in the topological blueprint of cancellous bone can be found between the groups and between the anatomical areas of the calcaneus. A more detailed presentation of the ITA values separately for all node types and for individual specimens can be found in the Supporting Fig. 5. One of the attributes of the skeletonization algorithm is the edge length. These values are plotted in the Supporting Fig. 6 and demonstrate no significant difference.

## Discussion

Foot binding is a unique historical phenomenon in which the anatomy of the foot, refined over several million years of human evolution, was intentionally altered for cultural and social reasons. As follows from interviews with the last survivors of the foot‐binding procedure—who are now around 90 years old—the extent of their adaptation allowed for sufficient independence. Some of the bound‐foot females reported that they were very physically active (dancing, bicycling, or bowling), in addition to most being routinely involved in agricultural labor.^16^ The reported active lifestyles, in the context of reduced shock‐dampening capacity of the feet, apparently indicate that effective biomechanical compensation took place. For example, accentuation of the lumbar curvature often described in historical texts as “splendid hips,” but likely being of a postural, rather than anatomical origin,^14^, ^15^, ^30^ could compensate for negative consequences of repetitive organ and joint impacts resulting from stiffness of gait. Moreover, from the close‐up photographs that exist of bound feet (Fig. 1 *B*, *D*, asterisk), it appears that the adipose pad in the heel could be unusually thick compared to its normal thickness in females, as reported to be about 12 mm.^31^ Compensatory thickening of the heel fat pad has also been observed to occur within 2 years following a foot trauma that reduces mobility of the ankle.^32^ It follows that soft tissue adaptation compensates for the deficiency of the shock‐dampening function of the bones of the foot. Thickening of the adipose pad, altered posture and gait likely contributed to shock‐dampening as a collective compensatory mechanism. Despite the effective compensation for the diminished shock‐dampening function in the bound‐foot individuals, certain characteristics of the three‐dimensional organization of the calcaneus differ significantly from the norm.

### Absence of normal biomechanical input precludes the development of the normal anisotropy pattern

A preferred alignment of trabeculae in the direction of so‐called stress trajectories can be identified by eye on a bone cross‐section or on a radiograph, the classic example being the human proximal femur. However, the validity of interpretation of the trajectories in the proximal femur has been questioned, at least in the context of two‐dimensional structural study^7^ and from the perspective of bone tissue development.^9^ Nevertheless, as previously reported by Ryan and Krowitz^33^ using the example of the human proximal femur, cancellous bone in infancy is characterized by a very uniform (isotropic) and dense three‐dimensional microarchitecture. With the onset of independent walking in a toddler, two processes are observed that happen in parallel: the total number of trabeculae decreases, whereas the anisotropy index increases. In response to function, certain trabeculae acquire pronounced preferred orientation, forming stronger texture, and some trabeculae become redundant and eventually get eliminated. This is apparently a normal process of functional adaptation of the nonspecialized default structure.^34^, ^35^, ^36^, ^37^

Here we show that in the normal foot the anisotropy index varies along the axis of the calcaneus in a recognizable fashion: the highest degree of anisotropy is observed in the vicinity of the Achilles tendon and plantar fascia attachments, and also near the subtalar articular surface of the calcaneus. Thus, the anisotropy pattern is indeed established by the action of recurrent biomechanical stimuli, such as tension and compression. Here, in the exceptional example of abnormal loading of bound feet, the mechanical inputs of the Achilles tendon and plantar fascia are eliminated. Consequently, a recognizable pattern of the degree of anisotropy does not develop in the bound‐foot calcaneus because there is no consistent mechanical input that would cause a predictable morphological output.

### Abnormal cancellous bone texture reflects the nonphysiological function of the bound‐foot calcaneus

We assume that the normal process of trabecular adaptation involves augmentation of the trabeculae aligned with the major stress directions and the increase of anisotropy, and this assumption leads to the following premises: first, anisotropy cannot develop in the absence of persistent and oriented stress stimuli. Second, the local anisotropy of cancellous bone reflects the diversity of the stress orientations: a narrow range of loading vectors results in a narrow range of orientations of the anisotropic “stress trajectories.” Interestingly, for the normal group, the degree of anisotropy is the highest where the direction of loading is the most uniform (ie, the direction of the tendon/fascia pull that are anatomically predetermined). Here, the degree of anisotropy was slightly lower in the talocalcaneal joint area, where the direction of loading is generally vertical but normally varies in the range of about 30 degrees.^13^, ^18^ In contrast, in the bound foot, a somewhat higher degree of anisotropy is locally present in the body of the calcaneus, and the lowest anisotropy is found distally in the tuberosity area. Thus, a curious inverse pattern is formed along the axis of the bound foot calcaneus, namely a relatively higher anisotropy in the proximal and central areas is observed, and declines distally. Biomechanically, the calcaneus of the bound foot functions as a rigid extension of the lower leg. Intriguingly, its peculiar anisotropy trend resembles the anisotropy pattern characteristic of most long bones. It can be observed in a longitudinal cross‐section of a long bone that the high anisotropy of the tubular diaphysis gradually decreases via coarse longitudinal trabeculae toward finer trabeculae throughout the flaring metaphysis and toward the finest trabecular network of the subchondral area,^38^ because it can be seen in a longitudinal long bone cross‐section. Hence, the calcaneus of the bound foot not only functions as a lower leg extension, but also adopts a similar anisotropy trend.

### Preserved fabric topology presumably is a determinant of mechanical competence of cancellous bone

The focus of the novel ITA analysis is different from conventional analyses of cancellous bone texture. First, regardless of how thick or thin trabeculae are, only their axes are identified and the three‐dimensional geometry of the connections is considered. Second, in the three‐dimensional trabecular fabric comprising hundreds of thousands of trabeculae, the configuration of their connections is analyzed statistically. The distribution of the ITA values shows two principal parameters: the most typical configuration of a connection of several trabeculae—the mean ITA; and the uniformity of such connections throughout the fabric—the width of the distribution. To illustrate; in the framework of a generic multistory building, the predominant angle between the elements will be 90 degrees (as walls and floors are expected to connect at right angles), and this angle distribution will be extremely narrow (all essentially 90 degrees). In a hexagonal bee honeycomb, the most common angle between connected elements would be 120 degrees, but, as in every biological system, the honeycomb accommodates a certain extent of irregularity, such as a slight variation of the honeycomb cell size, or accidental appearance of five‐edged or seven‐edged cells; thus, the distribution of the angles in a honeycomb normally would be 120 degrees plus or minus a certain angular value.^39^ Cancellous bone, being more complex than human buildings and bee honeycombs, first includes nodes of different (primarily low) valence, organized into a space‐filling three‐dimensional fabric. Second, cancellous bone is constantly remodeled by local resorption and apposition of bone. As a consequence of these factors, the ITA distribution is inevitably broad, but yet highly reproducible among different individuals and among various anatomical locations. Of note, local thickening or thinning of individual trabecular elements does not affect the ITA value as long as the three‐dimensional orientation of these trabeculae does not change.

The ITA parameters describe the basic blueprint of cancellous bone, according to the principles of the minimal fabric complexity and the maximal volume spanning. Together, these topological principles should allow for the combination of the lowest metabolic cost (prevalence of simple nodes) and the highest stability of the three‐dimensional framework (maximal spread of the connected trabeculae in three dimensions). The observation that the ITA parameters of bound and normal foot calcanei are nearly identical, and the fact that in both biomechanically different scenarios the bone tissue integrity is preserved (ie, no fractures reported), indicate that the ITA‐based topological characteristics of cancellous bone and its mechanical competence can be related. The future study of the topological blueprint in the developing cancellous bone of growing individuals, and in cases of skeletal pathology, will help to establish whether there is a causal relationship and whether cancellous bone strength is provided for by the optimized topology.

To summarize, the three‐dimensional arrangement of cancellous bone in the normal human calcaneus is a highly‐performing, anisotropic assembly that is essential to facile locomotion. Importantly, the foot as a whole adds shock‐dampening capacity. The loss of this shock‐dampening capacity in bound feet is compensated for by multiple mechanisms, such as smaller gait strides, altered weight distribution, and possibly even thickening of the fat pad of the heel. The calcaneus in the bound foot is a unique example where a normal anisotropic structure fails to develop, and a deviant anisotropy pattern forms. As opposed to the physiologically high anisotropy of the normal calcaneus specifically in the tuberosity region, in the bound foot a minimal anisotropy is observed at this site. Surprisingly, it appears that even under the abnormal loading conditions that occur in the bound‐foot calcaneus, the basic topological blueprint of trabecular bone fabric remains unaltered to preserve mechanical competence (ie, withstanding loading without fracture). Thus, we suggest that the lower degree of anisotropy, along with the intact basic topological blueprint identified by our ITA analysis, are the default characteristics of the trabecular bone tissue from which functional adaptation may proceed.

## Disclosures

All authors state that they have no conflicts of interest.

## Supporting information

Supporting Figure S1.
