# Supplementary material for: Functional Adaptation of the Calcaneus in Historical Foot Binding
Source: J Bone Miner Res. 2017 Jul 6;32(9):1915–25. doi: 10.1002/jbmr.3185 (PMC5603983; doi:10.1002/jbmr.3185)
Supplement: Supplementary file 1 — Supporting Figure S1. [file JBMR-32-1915-s001.docx]

**Functional adaptation of the calcaneus in historical foot binding. Supplemental Materials**

Dr. Natalie Reznikov^1^*, Dr. Carina Phillips^2^, Martyn Cooke^2^, Dr. Amin Garbout^3^, Dr. Farah Ahmed^3^, Prof. Molly M. Stevens^1^

**Affiliations**

^1^ Department of Materials, Department of Bioengineering and Institute for Biomedical Engineering, Imperial College London, Prince Consort Road, London, SW7 2AZ, United Kingdom.

^2^ Imaging and Analysis Centre, Core Research Laboratories, The Natural History Museum, Cromwell Road, London, SW7 5BD, United Kingdom.

^3^ The Hunterian Museum, The Royal College of Surgeons of England, 35-43 Lincoln’s Inn Fields, London, WC2A 3PE, United Kingdom.

**Corresponding author:** N. Reznikov [n.reznikov@imperial.ac.uk](mailto:n.reznikov@imperial.ac.uk); [naoree@gmail.com](mailto:naoree@gmail.com);

tel.: +44 7840027812

Materials

**
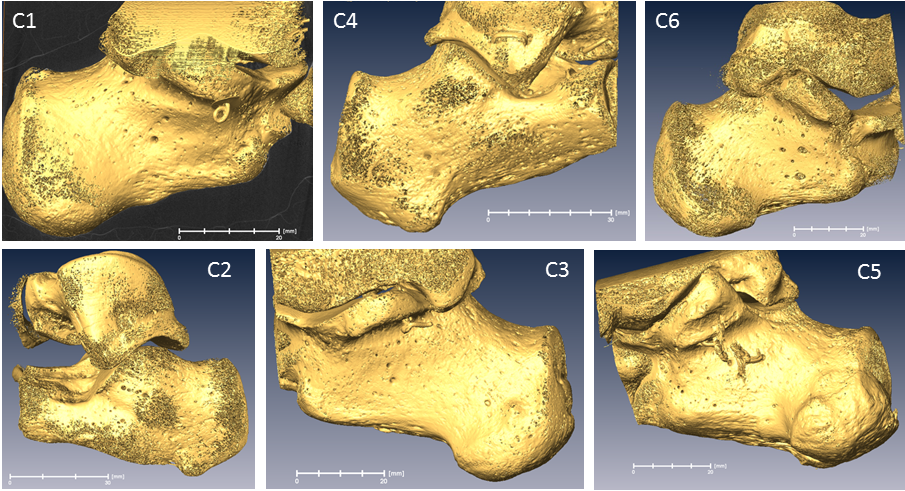
**

**Fig. S1. Control samples of the normal calcaneus, indexed as in the text.** Top row – right calcaneus, bottom row – left calcaneus. Scanning was performed with a pixel size 0.33 – 0.041 mm. All feet appear to be skeletally mature. Morphologically, the left foot C2 and the right foot C4 are very similar and could have originated from the same individual, but the following the texture and ITA analysis of the internal architecture they were found as close to each other as to all other normal samples.

Methods

**
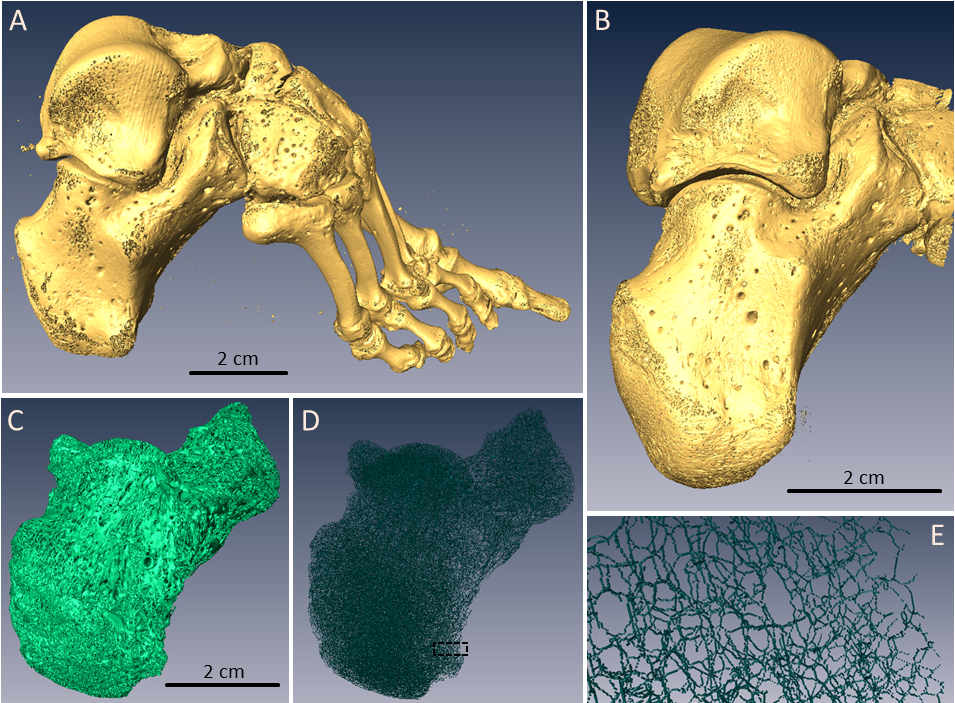
**

**Fig. S2. Imaging and analysis of foot specimens.** (**A**) Complete bound-foot specimen, pixel size *ca*. 80 µm; (**B**) area of interest (calcaneus), pixel size *ca*. 40 µm; (**C**) labelling of the trabecular interior of the calcaneus by manual exclusion of the compact cortical bone shell followed by automated global thresholding. Conventional analysis of trabecular bone texture was carried out using 3D rendering of the trabecular interior. (**D**) Digital skeletonization, where every trabecular element is replaced by a one-pixel-thick branch and the branches are connected at “nodes”; (E) enlarged portion of the skeletonized fabric, as indicated by the black dashed frame in (D). Topological analysis of the 3D fabric was carried out by investigating the manner in which adjacent trabeculae connect to each other.

Supplemental results below illustrate the parameters that did not display considerable difference between normal and bound foot samples.

**
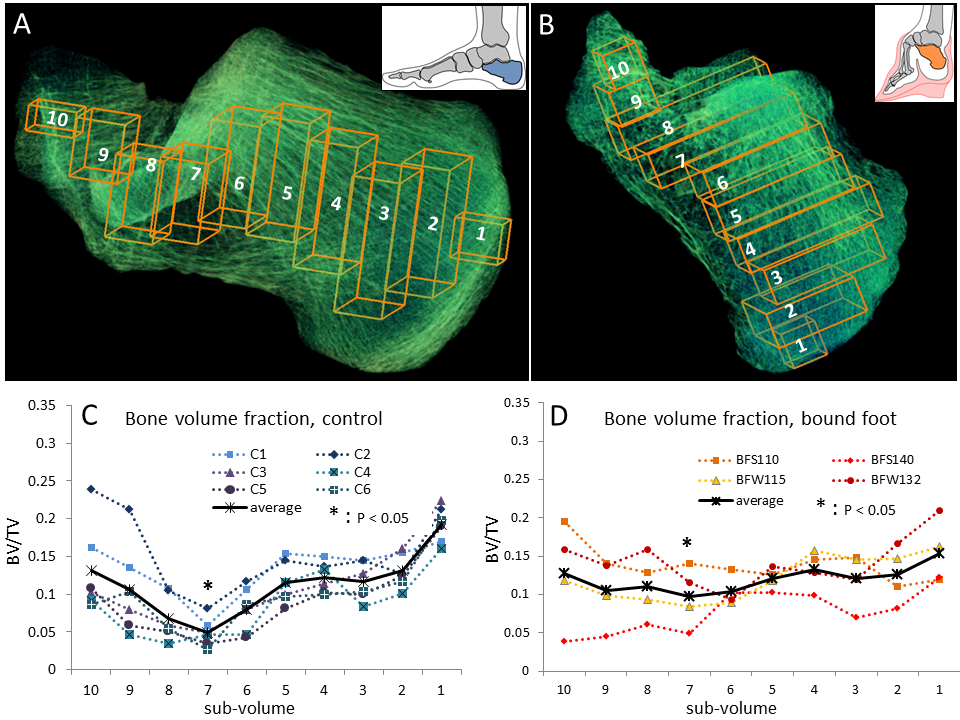
**

**Fig. S3. Ten cropped sub-volumes along the longitudinal axis of the calcaneus** (A and B) were used for estimating the general trend of the trabecular volume fraction parameter along the anatomical axis (C, control calcaneus; D, bound-foot calcaneus). Bone volume fraction was calculated for each sub-volume and plotted as individual measurements against the sub-volume number. Average values are plotted in black. Asterisk indicates the measurements with p<0.05 (two-tail unpaired T-test).

**
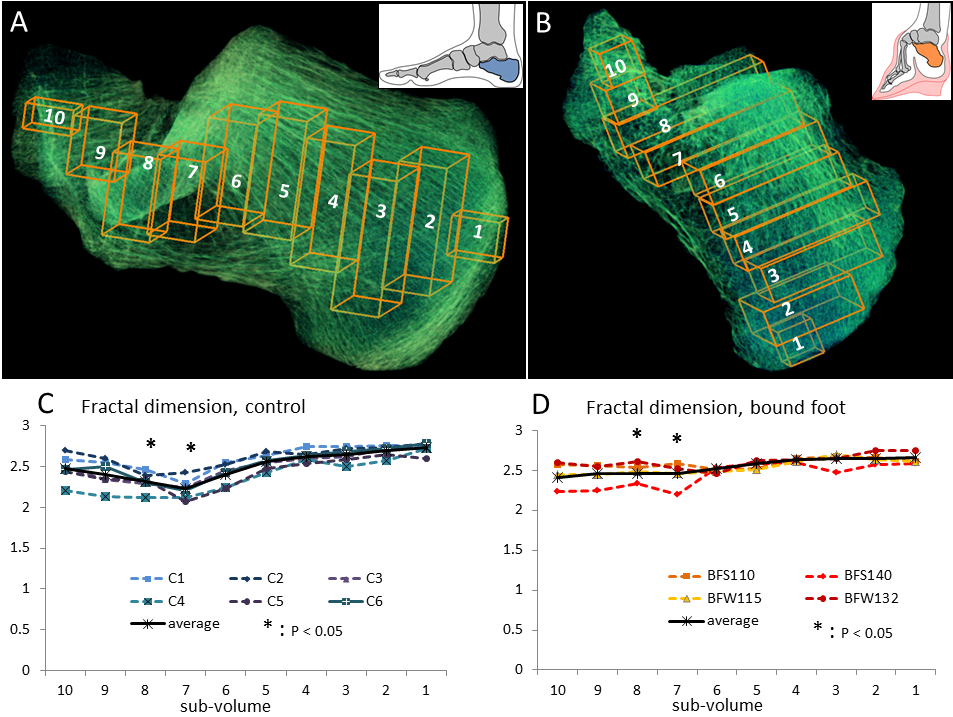
**

**Fig. S4. Ten cropped sub-volumes along the longitudinal axis of the calcaneus** (A and B) were used for estimating the general trend of the fractal dimension parameter (C, control calcaneus; D, bound-foot calcaneus). The fractal dimension was calculated for each sub-volume and plotted as individual measurements against the sub-volume number. Average values are plotted in black. Asterisks indicates the measurements with p<0.05 (two-tail unpaired T-test). Of note, the elimination of redundant trabeculae in a consistent fashion is manifested as a slight drop of the fractal dimension of cancellous bone in the normal calcaneus in the area where BV/TV is also the lowest (compare with the trend line in Fig. S3, sub-volumes 7 and 8).

**
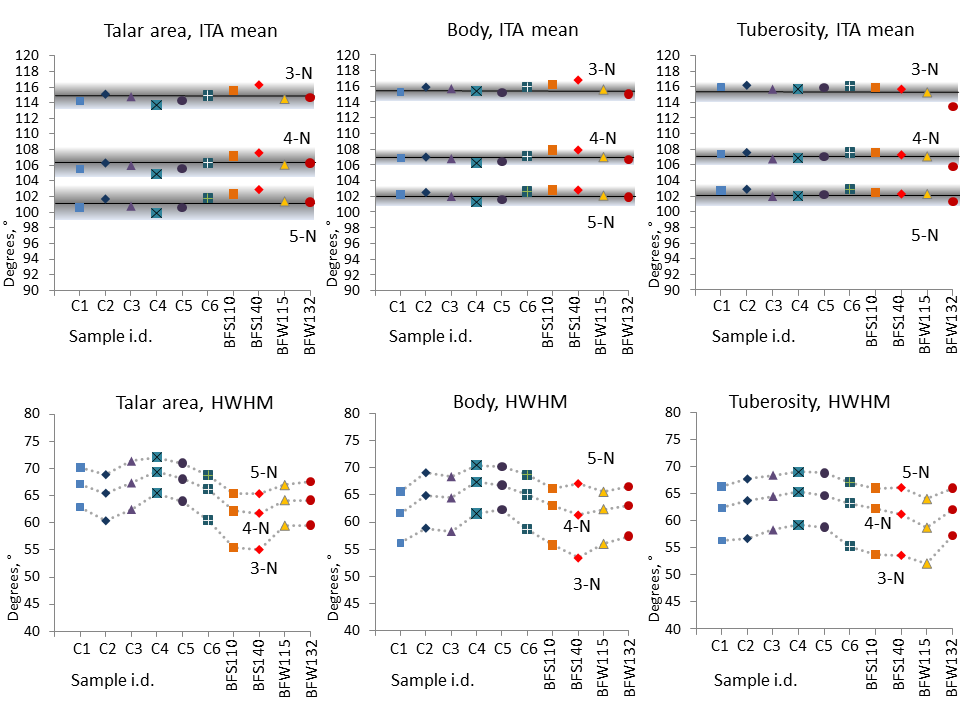
**

**Fig. S5. ITA distribution mean values and distribution width per anatomical area per individual.** The top row shows the mean ITA values in degrees (vertical axes); the complexity of nodes is labeled on the graphs as 3-N, 4-N and 5-N. The shaded bands in the top panels show variation (1 standard deviation) amongst the individuals. Note the high reproducibility of the ITA mean values. The bottom panels show the width (half-width at half-maximum, HWHM) of each ITA distribution in degrees. The top series stands for the HWHM of 5-N nodes, middle series is 4-N nodes and bottom series is 3-N nodes. Interestingly, the higher the ITA mean value, the narrower the ITA distribution: compare C4 and BFS140 ITA mean and HWHM in all anatomical areas. The general trend is that the higher ITA values that closely approximate the symmetrical 3D shapes’ values (*i.e.* 120°, 109.5°) are accompanied by narrower HWHM, indicating a more uniform space-filling fabric architecture.

**
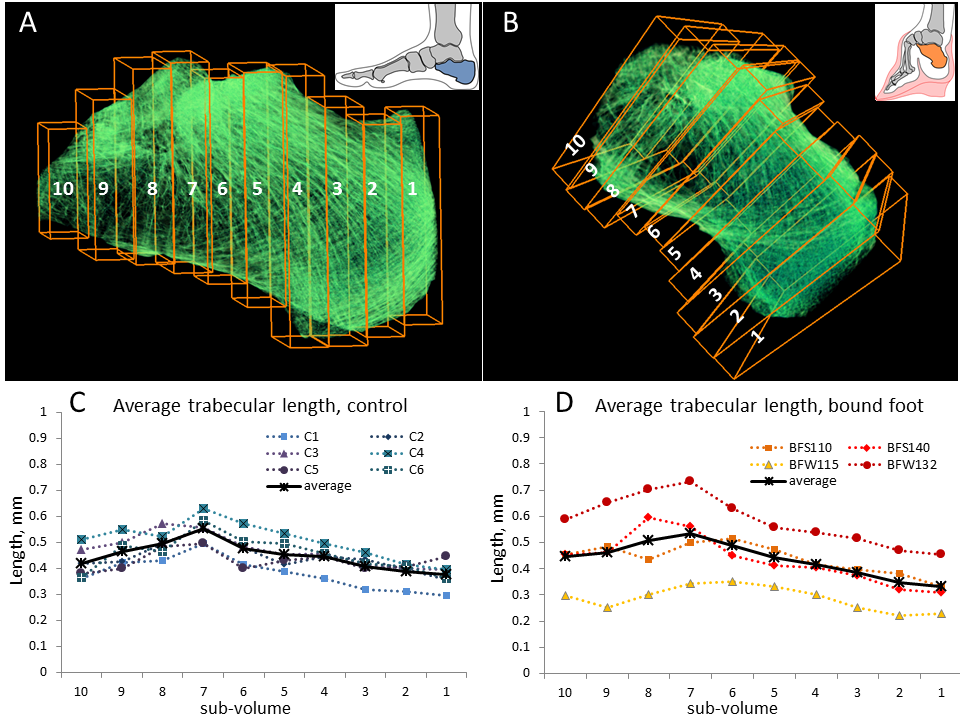
**

**Fig. S6. Ten cropped sub-volumes along the longitudinal axis of the calcaneus** (A and B) were used for estimating the general trend of the mean trabecular length (C, control calcaneus; D, bound-foot calcaneus) along the anatomical axis. Average trabecular length was calculated for each sub-volume and plotted as individual measurements against the sub-volume number. Average values are plotted in black.
